# Integrating a problem-solving intervention with routine care to improve psychosocial functioning among mothers of children with sickle cell disease: A randomized controlled trial

**DOI:** 10.1371/journal.pone.0252513

**Published:** 2021-06-09

**Authors:** Monika R. Asnani, Damian Francis, Jennifer Knight-Madden, Susan Chang-Lopez, Lesley King, Susan Walker

**Affiliations:** 1 Caribbean Institute for Health Research, The University of the West Indies, Kingston, Jamaica; 2 School of Health and Human Performance, Georgia College, Milledgeville, Georgia, United States of America; Public Library of Science, UNITED KINGDOM

## Abstract

**Objective:**

To assess the feasibility of a problem-solving skills training intervention in improving psychological outcomes in mothers of infants with sickle cell disease (SCD).

**Design and methods:**

This parallel randomized controlled trial recruited 64 babies with SCD, 6 to 12 months of age, and their mothers. Baseline measurements assessed mothers’ coping and problem-solving skills, depression, and parental stress before random assignment to intervention or control groups (n = 32 each). Problem-solving skills intervention was delivered through 6 monthly sessions, when babies attended for routine penicillin prophylaxis. All measurements were repeated for both groups at the end of the intervention period. Intention to treat analysis used repeated measures mixed models with the restricted estimation maximum likelihood approach.

**Results:**

The problem-solving intervention had no significant effect on mothers’ problem-solving skills (adjusted treatment effect: -1.69 points (95% CI:-5.62 to 2.25)), coping behaviours (adjusted treatment effect: 0.65 points (95% CI:- -7.13 to 8.41)) or depressive symptoms (adjusted treatment effect: -0.41 (95% CI: -6.00 to 5.19)). It reduced mothers’ level of difficulty in managing stressful events by 9.5 points (95% CI (-16.86 to -2.16); effect size: 0.21 SD). In the subgroup of mothers at risk of depression (n = 31 at baseline), the intervention reduced depression scores with treatment effect of 10.4 points (95%CI: -18.83 to -1.88; effect size: 0.67 SD).

**Conclusion:**

This problem-solving skills intervention study suggests feasibility and possible efficacy in improving some maternal outcomes. Further refinement and culturally appropriate adaptations of the intervention could lead to stronger effects.

## Introduction

Sickle cell disease (SCD) has been declared a global public health problem by the United Nations and World Health Organization [1], with more than 300,000 affected infants born annually [2]. SCD is a public health concern in Jamaica where it is the most common genetic disorder and approximately 1:150 babies are born annually with the condition. Whereas newborn screening (NBS) for SCD has been conducted in parts of the island sporadically over the last 40 years, universal island wide screening has been achieved since 2015.

The morbidity and mortality burden of SCD can be great especially in infancy and early childhood. Recommendations are that babies diagnosed with SCD are integrated into a healthcare facility for close monitoring and to access interventions, such as parental education and pneumococcal prophylaxis, which have significant benefits [3]. The burden of SCD affects not only the children, but also their caregivers and families. Parents may experience various emotions such as denial, sadness, guilt and stress when their newborn is diagnosed with SCD. Along with the emotional impact of their child’s illness, they are required to make many changes to their lifestyle to fulfill the demands and responsibilities that come with managing the child. Parents and caregivers play a crucial role in ensuring their child’s good health, positive adaptation to their illness, and improving their daily functioning [4].

Parental stress has been shown to be high in parents and caregivers of children with a chronic illness [5], and can worsen both child and adult outcomes [5, 6]. Higher stress in mothers of children with diabetes has been shown to be related to lower maternal positive psychological well-being [7], higher maternal anxiety and depression [8], and suboptimal glycaemic control in the child [5]. Higher parental stress in parents of children with cancer is correlated to lower parental adjustment [9]. Greater parental stress may be associated with greater perceived child vulnerability, less child self-care behaviours and greater parental overprotection in children with various chronic diseases [10, 11]. Within SCD, increased parental stress has been correlated to lower quality of life of their children [12], higher disease severity and higher levels of healthcare utilization [13]. Additionally, parental stress has been identified as a significant risk factor for poor early cognitive development in young children with SCD [14]. Higher levels of parental education [15], income and family functioning [13] have been shown to be associated with lower parental stress and better problem-solving skills in parents of adolescents and school-age children.

Various behavioural and family based interventions have been considered in reducing parental distress and improving their adjustment to their child’s chronic illness such as cancer [16] and general medical conditions [4]. A systematic review and meta-analysis of interventions [4] has shown that pooled psychological therapies (all data pooled across treatment types) may have a positive effect on parent behaviours. Problem-solving therapy (PST) in particular shows promise in its reported association with improvements in parental mental health and behaviours [4, 6]. PST, which is based on a cognitive behavioural therapy model, aims to enhance coping with minor and major stressors [17]. It has been shown to be effective in persons with depression, and caregivers of children with chronic illness [4, 6, 18]. Enhanced problem-solving skills in parents of children with SCD have shown an association with improvements in the child’s quality of life [15]. A family based, group problem solving intervention to improve school functioning in children with SCD was found to be acceptable but no intervention effects were reported [19]. However, one of the benefits identified in this study was having parents of children with SCD participating and sharing together in the intervention session. Additionally, PST has been successfully delivered in primary care settings by both practice nurses and general practitioners [18, 20], as well as by community nurses and by providers with no formal mental health training [21, 22]. In this pilot study, we assessed the feasibility and potential efficacy of a problem-solving skills training intervention, aimed at improving psychological outcomes in mothers of children with SCD delivered during routine monthly clinic visits to small groups of parent/s of babies with SCD by nurses expert in managing SCD.We hypothesized that this parenting intervention would improve parental stress, coping, depression and problem-solving skills in mothers of infants with SCD.

The overall study also examined the effects of building parenting skills (to improve interaction with their children) on children’s development (fine and gross motor development, cognition and language). This evaluation will be reported elsewhere.

## Materials and methods

This was a parallel (1:1 allocation), randomized controlled trial with mothers of infants diagnosed with SCD through new-born screening conducted from March 2015 to September 2016. Ethical approval for the study (S1 Appendix) was granted by the University of the West Indies (UWI) Ethics Committee (ECP # 243, 13/14; approval obtained October 21, 2014) and Southern Regional Health Authority of Ministry of Health, Jamaica Ethics Committee (approval obtained March 3, 2016) before any study procedures began. As enrollment numbers were slow and not expected to meet target during study period allowed by the funders, the study was expanded to the southern region of the island where some of the newborn screening for SCD occurs as well. Enrollment in southern region began only after ethical approval was obtained from their health authority.

Two changes were made to the initial protocol. One was to to alter the age group of children in the recruited dyads from 6–9 months to 6–12 months. This is for logistical reasons to ensure that the study can recruit sufficient number of participants to complete the project within time frame. The second was to add some questions that allow us to study social support, and Depression (using the CES-D scale). Both changes were made prior to 1^st^ subject enrollment. The trial was registered with ClinicalTrials.gov (Identifier: NCT02394899) on March 20, 2015 and 1^st^ enrollment occurred on March 25, 2015. The authors confirm that all ongoing and related trials for this intervention are registered. (For Consort checklist, please see ‘S1 Checklist’).

### The study population

All mothers of children aged 6–12 months with severe SCD genotypes (Hb SS disease and Hb Sβ^0^ thalassemia) attending the clinic at the Sickle Cell Unit (SCU) at the UWI, and Mandeville Regional Hospital (MRH), Jamaica were eligible and informed of the study and given an opportunity to participate. Every eligible baby aged 6–12 months of age attending the SCU and MRH during the study period was enrolled in the study. There were no refusals.

The SCU is located in Kingston, the capital of Jamaica and is partly supported by the government of Jamaica whereas MRH is a government hospital also located in an urban area and serving surrounding rural areas. The second site contributed only a small number (n = 7) persons to the study and no site differences were assessed. However, all procedures were performed in the same way and by the same study staff.

Once the written, informed consent had been obtained from the subject, then baseline measurements (T0 measurements) were completed by the study coordinator. A random numbers table, computer generated by an independent staff statistician, was kept centrally. Once baseline measurements were completed, the study coordinator telephoned the central site for the next assignment based on the random number table and informed the subject of her allocation group. The control group received usual clinical care which includes routine health maintenance, general counselling about SCD, its various presentations and complications, as well as discussions about management of child’s SCD at every visit to the study site. Each mother received the equivalent of US$ 17 to cover transportation and a snack for the baby at each visit.

### Procedures

A study coordinator, who was not involved with clinical care of persons with SCD, recruited the parent either at the clinical facility while they waited for their infant to be seen by the physician or via telephone contact. The purpose and the procedures for the study were thoroughly explained to them and all queries addressed fully. The study included 8 study visits for each mother: an entry visit (T0), 6 monthly visits when routine care or routine care + intervention were provided and an exit visit (T1). The monthly visits were booked at 4 weeks intervals but could be delayed up to 2 weeks. If a mother and baby dyad could not attend a visit (intervention or control) within two weeks of the scheduled visit, then that was deemed a ‘missed visit’. Assessments were done at baseline (T0) and exit visit (T1) which occurred 1–2 weeks after last intervention visit. Assessments included: socio-demographics including social support; parent’s problem-solving skills; parental stress; parental coping and parental depression. All testing and visits were conducted at the participants’ main clinical care site.

All questionnaires (in English) were interviewer administered in private surroundings by trained testers who were blind to the randomization arm of the participant. These testers were trained in questionnaire administration and other measurements, but they were not involved in any other aspect of the project. The questionnaires were pilot tested prior to commencement of enrolment into the current study and this pilot testing was conducted with mothers who were not subsequently included in the current study. The time for completion of assessments and questionnaires was 60–90 minutes.

### Instruments

#### Socio-demographics

Socio-demographic information was collected using an interviewer administered questionnaire. Information collected included marital status of mother, religion, number of household possessions, mother’s education, mother’s employment, and the presence of father or father figure in the home. Information on the mother’s occupation was collected based on current employment and coded according to the Jamaica Standard Occupational Classification 1991(JSOC-91). Occupations were categorised into three groups: highly skilled, skilled and semi-skilled/unskilled.

#### Social Problem-Solving Inventory–Revised: Short Form (SPSI-R:S)

This is a 25-item instrument for use in persons over 13 years of age [23]. It assesses problem- solving capacity on a 5-point Likert scale and higher total scores indicate higher levels of more constructive problem-solving skills [15]. It has been used in studies across a number of countries such as United Kingdom, South Africa and Japan and across a variety of clinical conditions including cancer, diabetes mellitus, and caregivers of persons and children with chronic diseases [24–26] including SCD [15]. To our knowledge, it has not been used in previously used in Jamaica and inter-item reliability (Cronbach’s alpha) for the SPSI-R:S total score was 0.79 in our study.

#### Pediatric Inventory for Parents (PIP)

This inventory [27] examines perceived stress in parents of a child with a chronic illness. It is a 42-item instrument scored on a Likert scale, and has questions regarding how often a particular event has occurred (Frequency) and how difficult that event (Difficulty) was for the respondent. Scores are calculated in 4 sub-domains (Communication, Medical care, Emotional Disturbances, and Role Functioning) and then totalled to give a total difficulty and a total frequency score with higher scores indicating higher levels of stress in each domain. The scale has been used widely across USA, Netherlands, Spain and China and in numerous populations including parents/caregivers of children with cancer, diabetes, and sickle cell disease. Inter-item reliability was 0.91 for the ‘Difficulty’ subscale and 0.84 for the ‘Frequency’ subscale in our study.

#### Coping Health Inventory for Parents (CHIP)

This 45-item measure [28] aims to measure how parent/s or caregiver/s cope with managing a child with a chronic illness. It is scored on a Likert scale and a total score calculated. Higher scores reflect greater use of coping behaviours. It has been used widely across many countries such as USA, Mexico, Iran, and Korea but not in Jamaica. Inter-item reliability for the total CHIP score was 0.88 in our study.

#### Centre for Epidemiologic Studies Depression Scale (CESD)

This 20-item scale [29] measures symptoms of depression. It is scored on a Likert scale and a total CESD score is calculated as a sum of response to all items. The possible scores range from 0 to 60 with higher scores indicating presence of higher numbers of depression symptoms. The CESD has been used in Jamaica previously [30]. Inter-item reliability was 0.90 in our study.

### Intervention

#### Problem-Solving Therapy (PST)

Problem-solving skills therapy aims to empower patients or caregivers in attending to daily social and other challenges that might arise especially with the presence of a chronic illness. The approach is based on cognitive behavioural therapy and has been shown to be of use in primary care settings [4, 18, 31, 32]. It encourages persons to use existing resources and skills to function better and find solutions to problems [20]. We adapted for our use in parents of a child with SCD a version of PST called ‘Bright IDEAS’ which is a problem-solving skills training intervention [25] that has been established as an effective intervention in mothers of children with cancer. Permission for use of this program was obtained from the developer. The stages of the training are: identification of the problem/s; generating possible solutions; evaluating the options; implementing the preferred solution; and evaluating to see if the solutions were successful [6, 31]. The original ‘Bright IDEAS’ manual is written for parents of children with cancer and we made the appropriate changes to the manual for use in parents of children with SCD. These changes were minimal and were made mainly for the role play sessions where examples included problems that mothers of babies with sickle cell disease might experience. Even though the original program has been mostly used in individual sessions, the developers do state that it is useful to be delivered in group settings which is how the sessions for this study were conducted. In fact, bringing parents together in a group setting to deliver another family-based problem solving intervention has been found to be of benefit [19]. A group based intervention using PST has been used to promote adjustment in caregivers and children with congenital heart disease [33]. The intervention was considered feasible as mothers bring their babies for penicillin prophylaxis every 4 weeks and the same group of mothers returned at the same time thus facilitating the monthly intervention sessions.

The intervention was delivered by three SCU clinic nurses who are experienced and routinely provide clinical care as well as genetic and disease counselling to persons and families with SCD at the Sickle Cell Unit. They underwent an initial period of training in PST with the study investigators. This training was delivered through 5 sessions conducted over 2 weeks and totalling 9 hours. It included discussions of the intervention, use of the interventionists’ manual and role-playing. The mothers were taught a process of problem-solving with reference to problems they identified themselves, both general everyday problems as well as specific problems which arose while parenting a child with SCD. The study coordinator also attended these sessions. The problem-solving therapy followed the Bright Ideas manual, which has an instructor and patient specific version, and details the steps to be taken to solve daily problems related to the illness, work, home etc. Each session followed a standard protocol. To assess fidelity, a checklist was developed with items that measured the interventionists’ implementation of the various steps of the intervention. The checklist had 3-point ratings on items that monitored how well each interventionist showed empathy during the intervention, allowed the mother to be actively involved in the discussion, and facilitated a thorough understanding of the problems as well as possible solutions for these problems being identified by the mother. These were completed by the study coordinator who observed the interventionists at every third visit to monitor the consistency of delivery. The median rating for the sessions was 12 (maximum:12; interquartile range: 11.25–12) suggesting good completion of the session. The coordinator gave supportive feedback at end of each observed session. The first two sessions were also observed by the study investigators. The group intervention had 3–5 mothers per 45 minutes session and was conducted by two clinic nurses at a time. A total of six intervention sessions were planned for each group.

All mothers in the treatment arm also received an intervention to build their skills in promoting their child’s development that was delivered at the same session. The babies were therefore present at each intervention visit with their mother. The parenting skills intervention used videos, discussion, demonstration, and practice of activities, and has been used previously in Jamaica [30]. Interventions were conducted in a private room, away from the general clinic, to avoid leakage of the intervention to the control group. The control mothers attended individual routine clinic visits on days that the intervention groups were not being held. At the end of the study, control dyads were exposed to all intervention materials.

### Process assessments

A process audit was conducted at the end of the study. A random sample of mothers in the intervention group were invited for an individual in-depth interview with an independent interviewer and interviews continued till saturation was achieved. Nurses who delivered the intervention were also interviewed. The nature of the interview was explained and permission obtained before interviews were conducted. The purpose of the interview was to assess their experience with the intervention, what they liked and/or disliked about the intervention, and any recommendations they had to improve the intervention for future utility. Using a grounded theory approach [34], a coding list was generated inductively where transcripts were reviewed thoroughly and codes were assigned as concepts became apparent. Thereafter, thematic analysis [35] was applied to unifying and recurrent ideas that emerged from the conceptual codes generated. Interviewing, transcription and analysis was conducted by an independent research assistant who was not otherwise involved in the trial.

### Statistical analysis

To our knowledge, no problem-solving skills training interventions have been conducted in Jamaica in the past. Therefore, the sample size was determined for the impact on the child development outcome of the trial as local data were available from a Jamaican trial of a home visiting parent intervention with similar content integrated into primary care that achieved an effect size of 0.8 SD [36]. The current intervention had fewer numbers of contacts with mothers hence it was hypothesized that an effect size of 0.67 SD would be appropriate for sample size estimation. A sample comprising 35 children per group was estimated to achieve 80% power and a type I error rate of 0.05 in detecting the hypothesized effect. During the study period 64 mother child pairs were enrolled with 32 pairs in both the intervention and control groups, which reduced the power of the study to 79%.

Analyses were performed based on intention to treat principles using the statistical software STATA 14.2 (StataCorp. 2015. Stata Statistical Software: Release 14. College Station, TX: StataCorp LP.).

The primary outcomes were 1) Parental stress, depression and coping, as measured by the Pediatric Inventory for Parents, CES-D, and Coping Health Inventory for parents, respectively; and 2) Parental problem solving skills as measured by the Social Problem Solving Inventory. Association between explanatory variables and maternal outcomes at baseline were examined using ANOVAs. Independent covariates for intervention effect models were chosen based on a conservative significance level of p<0.2.

Intervention effects on outcomes between T0 and T1 were analysed using repeated measures mixed models with the restricted estimation maximum likelihood approach [37]. Effect sizes were calculated using Cohen’s D which indicates the standardised difference between two means.

Using established criteria for CES-D scores, persons were dichotomously categorized into ‘high-risk’ (CESD score at baseline of 16 points or more) and ‘not at risk’ groups. As the CES-D is able to categorize persons this way, and the fact that the current intervention is known to have positive effects in persons with depression, post-hoc subgroup analysis was conducted to examine the impact of the intervention on participants considered at ‘high risk for depression’.

## Results

**Fig 1:**
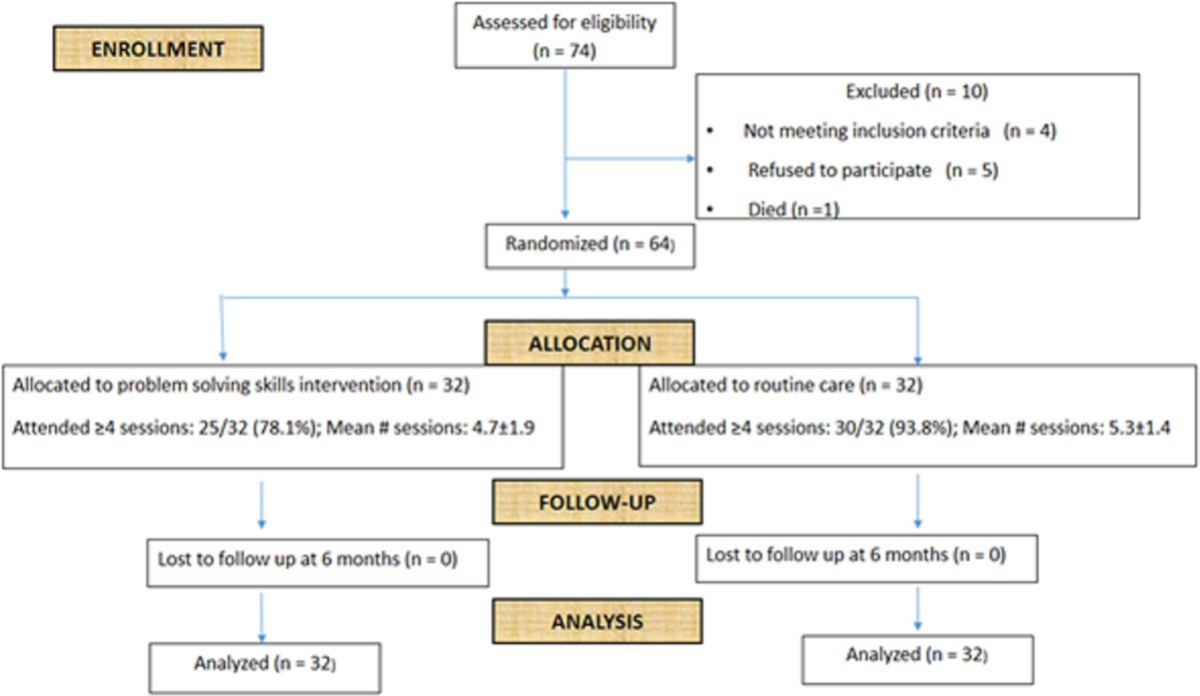
Consort diagram showing trial participant flow.

A total of 74 mothers were approached and assessed for eligibility. Of these, 10 were excluded and hence 64 mothers were recruited to the trial. The mean age of mothers in the intervention group was 28.7 years (±6.4 years) and 28.8 years (±5.4 years) in the control group. The flowchart given in Fig 1 describes the trial flow. Nine participants (14.1%; intervention = 7 and control = 2) had 3 or less visits to the clinic (of a planned 6 visits) during the study period. Most missed appointments were due to their baby being hospitalised from complications of SCD.

The majority of the mothers in the study were in a marital (20.3%) or common law relationship (48.4%) and reported their highest level of education as secondary (71%). Few homes (9.4%) did not have the child’s father or a father figure present. Approximately one half (45.3%) of households were in the lowest tertile of household possessions and 1 in 3 mothers reported having high level of social support. Sixty percent of mothers in the trial had two or more live births; however, the majority (82.8%) reported having only a single (current) child with sickle cell disease. Baseline comparison of intervention and control groups indicated that mothers receiving intervention had more household possessions compared to mothers in the control arm (p = 0.04). There were no other significant differences between groups at baseline (Table 1).

**Table 1:** Baseline maternal and home characteristics of control and intervention group.

	Control (n = 32)	Intervention (n = 32)	Overall (n = 64)
Mothers Age, years (mean, SD)	28.8, 5.4	28.7, 6.4	28.8, 5.9
Marital status, n, (%)			
Never married/single	8 (25.0)	12 (37.5)	20 (31.3)
Common-law	16 (50.0)	15 (46.9)	31 (48.4)
Married	8 (25.0)	5 (15.6)	13 (20.3)
Father or Father-figure present in home, %	29 (90.6)	29 (90.6)	58 (90.6)
First Child, %	12 (37.5)	12 (37.5)	24 (37.5)
Live births, %			
one	11 (34.4)	13 (40.6)	24 (37.5)
2–4 births	19 (59.4)	19 (59.4)	38 (59.4)
5 or more births	2 (6.3)	0	2 (3.1)
Births with SCD, %			
None	0	1 (3.1)	1 (1.6)
one	25 (78.1)	27 (84.4)	52 (81.3)
2 or more	7 (21.9)	4 (12.5)	11 (17.2)
Highest Education, %			
Grade 9 or lower	1 (3.2)	1 (3.1)	2 (3.2)
Secondary	22 (71.0)	23 (71.9)	45 (71.4)
Tertiary	8 (25.8)	8 (25.0)	16 (25.4)
Occupation category^+^, %			
Unskilled/Semi-Skilled	7 (58.3)	5 (33.3)	12 (44.4)
skilled	3 (25.0)	9 (60.0)	12 (44.4)
Highly Skilled	2 (16.7)	1(6.7)	3 (11.1)
Household possession (tertile)*, %			
lowest third	15 (46.9)	14 (43.8)	29 (45.3)
middle third	10 (31.2)	3 (9.4)	13 (20.3)
highest third	7 (21.9)	15 (46.9)	22 (34.4)
Social support, %			
lowest third	14 (43.8)	10 (31.3)	24 (37.5)
middle third	6 (18.8)	11 (34.4)	17 (26.6)
highest third	12 (37.5)	11 (34.4)	23 (35.9)
Sessions attended, %			
<4 sessions	2 (6.3)	7 (21.9)	9 (14.1)
4 or more sessions	30 (93.7)	25 (78.1)	55 (85.9)

^+^JSOC (Jamaica Standard Occupational Classification) among those employed

* p < 0.05.

### Association between outcomes and co-variates at baseline

ANOVAs were used to examine the relationship between explanatory variables and maternal outcomes as measured at baseline (T0). We found that the total coping score was associated with higher social support (F (2, 63) = 9.89, p<0.001) and maternal educational level (F (2, 63) = 2.39, p = 0.10). Being married was associated with lower depression score (F (2, 63) = 3.24, p = 0.05) and lower frequency of parental stress score (F (2, 63) = 2.77, p = 0.07). The difficulty handling parental stress score was higher with father-figure’s presence in the home (F (1, 63) = 2.13, p = 0.15), and was lower with higher social support (F (2, 63) = 2.48, p = 0.09), and being married (F (2, 63) = 2.60, p = 0.08). Problem-solving scores were higher with father-figure’s presence (F (1, 63) = 3.16, p = 0.08), greater social support (F (2, 63) = 3.59, p = 0.03), higher numbers of possessions (F (2,63) = 1.62, p = 0.21) and higher maternal education (F (2, 63) = 5.8, p = 0.005).

### Effects of intervention

The intervention resulted in no significant change to mothers’ problem-solving skills (adjusted treatment effect: -1.69 points (95% CI:-5.62 to 2.25)), coping behaviours (adjusted treatment effect: 0.65 points (95% CI:- -7.13 to 8.41)) or depressive symptoms (adjusted treatment effect: -0.41 (95% CI: -6.00 to 5.19)). It had a significant benefit of reducing mothers’ difficulty managing parental stress (total PIP difficulty score), with an adjusted treatment effect of -9.3 points (95% confidence interval (CI): -16.75 to -1.91); p value: 0.01) which is equivalent to a Cohen’s D effect size 0.21 SDs (Table 2). Post-hoc sub-group analysis of mothers ‘at high risk for depression’ (CESD ≥ 16; n = 31 at baseline) found that depression scores decreased significantly more in mothers in the intervention group (CESD scores: T0 (n = 15): 28.3±8.5; T1 (n = 15): 16.6±11.7) than those in the control group (CESD scores: T0 (n = 16): 25.8±7.0; T1 (n = 16): 24.4± 11.6) with a treatment effect of -10.4 (95%CI: -18.83 to -1.88; p value: 0.02) and Cohen’s D effect size: 0.67 SDs.

**Table 2:** Parental psycho-social outcomes measurements.

Outcome	Pre-Intervention (n = 32)	Pre-Control (n = 32)	Post-Intervention (n = 32)	Post-Control (n = 32)	Adjusted Intervention Effect (95% CI)	P-value
Coping Health Inventory for Parents (CHIP) Total Score^a^ (mean ± sd)	73.8± 19.5	72.6± 26.1	68.0± 21.0	67.9± 27.1	0.65 (-7.13 to 8.41)	0.87
Centre for Epidemiologic Studies Depression Scale (CESD) Total Score^b^ (mean ± sd)	15.9±13.4	17.2± 10.4	14.0± 10.3	15.7± 12.3	-0.41 (-6.00 to 5.19)	0.89
Post-hoc subgroup analysis CES-D scores* (mean ± sd)	28.3±8.5 (n = 15)	25.8±7.0 (n = 16)	16.6±11.7 (n = 15)	24.4± 11.6 (n = 16)	-10.4 (-18.83 to -1.88)	0.02
Pediatric Inventory for Parents (PIP)						
PIP Frequency Score^c^ (mean ± sd)	68.4±11.8	66.4± 12.6	62.2± 9.7	61.6± 15.0	-1.37 (-7.70 to 4.97)	0.67
PIP Difficulty Score^d^ (mean ± sd)	74.3± 20.3	68.5± 15.9	68.0± 16.4	71.8± 19.8	-9.33 (-16.75to -1.91)	0.01
Social Problem-Solving Inventory–Revised:Short Form (SPSI-R:S) Total Score^e^ (mean ± sd)	93.4± 11.3	88.8± 8.6	93.6± 10.6	91.6± 9.8	-1.69 (-5.62to 2.25)	0.40

Models adjusted for

^a^ social support, maternal education

^b^ marital status

^c^ marital status, social support

^d^ social support, father-figure presence, marital status

^e^ household possession, social support, father-figure presence, maternal education.

*: Post hoc analysis for mothers at high risk of depression (CESD ≥ 16; n = 31) at baseline.

#### Process audit

A total of 14 intervention mothers and all three nurses who delivered the intervention were interviewed. In general, participant mothers indicated that they found problem-solving helpful because they were able to use it in every area of their lives: not just with their babies. It also helped them to change their ways of handling problems that arise in childrearing. Though some mothers said that some of the language was not always clear, the nurses responded to their questions and some asked a friend about words they had difficulty with. Some mothers reported that they actually ended up using what they learnt during the sessions to help friends who were having their own problems. Mothers stated that they did not always find solutions to their problem using the problem-solving methods, however they said they would wait a while and attempt it again. Some indicated that it helped to improve their self-esteem. All participant mothers indicated that they would recommend the program to others who would want to be a part of it.

The nurses generally thought it was a useful training to conduct with mothers. However, some issues that could be improved were identified. They thought it took some time for mothers to be comfortable enough to identify problems in a group setting. Nonetheless, they mentioned that there were mothers who were more willing to discuss their problems and this encouraged others to open up also. Mothers were not keen to write down in the manual that they had been given the problems they identified between intervention visits. More role-playing and videos depicting problem-solving could also be helpful.

## Discussion

This study evaluated the benefits of a problem-solving skills therapy intervention on psychosocial outcomes among mothers of infants diagnosed with SCD. Mothers in the intervention group perceived less difficulty in handling stressful events post-intervention. In mothers determined to be at risk for depression there were significant improvements seen in the total depression scores associated with the intervention. There were no other benefits. The delivery of this intervention by existing healthcare staff during routine clinic visits of the patients makes it particularly attractive for application in even low resource settings.

The problem-solving skills intervention in this study appears to be efficacious in reducing both difficulty in handling stress and depression scores, and past studies also report that greater parental stress is associated with more symptoms of parental depression and anxiety [8, 38]. Interestingly, our baseline scores on both PIP difficulty and frequency scales are lower than studies have documented- such as in parents of children with diabetes [39], cancers [27] and sickle cell disease [12]. This difference might be present as most of the babies with SCD in the current study had not yet experienced complications associated with the disease and parental stress was therefore relatively low. Previous studies have reported that a problem-solving intervention can significantly lower parental stress and reduce depressive symptoms in parents of children with autism spectrum disorder [40] and lower depression in low-income mothers of premature babies [22].

The socio-economic profile of the study mothers was moderately high to high and intervention gains might be more significant and larger in participants who are socially more disadvantaged. Overall, educational attainment was high among mothers in the study when compared to national estimates [41] though other indicators of socioeconomic status such as household possessions and occupation appeared similar [30] or lower [42]. The literature suggests that outcomes such as problem-solving skills and parental stress levels being examined in this study are positively correlated with parental education, income and family functioning [15, 43, 44]. In our study, mothers had moderately high social support as well as the presence of father or father figure in the majority of homes. Several studies assessing caregiver health and wellbeing while caring for children with high levels of dependency showed that the social support provided by extended families decreases caregivers’ responsibilities and stresses, and attenuate caregiver burden [45–47]. Therefore, the presence of social support available to some of the caregivers in this study may have contributed to the small effects seen. The contribution of socio-economic factors and social support requires deeper understanding on how any modifications or adaptations being considered to the intervention will need to complement the mechanism by which these factors work to improve parental psychosocial health. The process should also be balanced with maintaining the rigor of the original designed and tested intervention [48]. The cognitive appraisals parents have of their child’s illness may moderate the relation between the stress they feel and other psychosocial experiences such as coping measures they adapt or experiences of depression/anxiety they face [5, 49]. Hence, results may improve if a component targeting parental resilience to enable more positive perceptions of their child’s illness is added to the intervention [49].

Tailoring interventions that work in other settings to suit one’s cultural and social environment can further improve their effectiveness [50]. One limitation identified through the delivery of the intervention was hesitancy on the part of some of the participants as one of their roles during the intervention- that of writing on the problem-solving steps in the manual- was unacceptable to them and so their engagement in the overall process could have been affected as a result. Though the intervention visits were conducted in English language by culturally and racially similar individuals, a redesign of the intervention manual such as shortening and simplifying the information given and reducing the need for them to document their experiences between intervention visits could improve its acceptability. The process audit though suggests mothers have positive perceptions of the PST intervention and its benefits. Increasing the intensity of the intervention (greater number and frequency of sessions) may improve benefits even though many studies have recommended use of 4–6 sessions as adequate for a PST intervention [18, 20, 22, 40] as increasing numbers beyond these may be unnecessarily repetitive. Additionally, providing individually focussed intervention, using other methods of delivery, and use of media such as videos showing examples of PST being used might also have led to stronger effects. Further testing of the intervention after more culturally appropriate adaptations and refinement may lead to larger improvements in outcome. There was good adherence to the intervention process and the supervision as was provided in this study might be necessary as without it the intervention may not have any impact. It is also possible that longer training and closer supervision of the interventionists could improve outcomes but there is added cost to this provision which could be a limiting factor. Any adaptations would need to consider demands on parents and healthcare resources to ensure feasibility within low- and middle-income settings.

There were low attendance rates at intervention visits for some children leading to limited improvement in outcomes as demonstrated in the study, although 75% of the sample had at least 4 of the 6 planned visits. The nature of SCD implies that young children will get sick and often require hospitalizations and other healthcare interventions. In the current study a number of missed or delayed intervention visits were due to the children’s acute illness episodes. In this case, the intervention might need some adaptation including a longer time period. The relatively small sample size is another limitation of the study. Furthermore, complications of SCD are relatively uncommon in the very young but rise as the infant gets older and therefore it might be of benefit to examine the long-term effects of this intervention. This study can be used as a pilot, feasibility study which can guide further tailoring and evaluation before wider-scale implementation.

In conclusion, the Bright IDEAS program appeared to have modest, but positive, effects on the psychosocial status of mothers of young children with SCD. The delivery of the intervention in a clinic setting where patients are seen at monthly visits allows for relatively straightforward integration into the services. Further modification is needed to improve the impact of the program before testing in a larger trial.

## Supporting information

S1 Checklist Problem-solving intervention trial consort checklist. (DOC)

S1 Appendix Problem-solving intervention trial protocol. (PDF)
