# Supplementary material for: Integrating a problem-solving intervention with routine care to improve psychosocial functioning among mothers of children with sickle cell disease: A randomized controlled trial
Source: PLoS One. 2021 Jun 9;16(6):e0252513. doi: 10.1371/journal.pone.0252513 (PMC8189456; doi:10.1371/journal.pone.0252513)
Supplement: S1 Appendix — (PDF) [file pone.0252513.s002.pdf]

Integrating a parenting intervention with routine care to improve parental psychosocial functioning and early developmental outcomes in children with sickle cell disease

Jennifer Knight-Madden, Susan Chang-Lopez, Monika Asnani, Lesley King, Susan Walker

Tropical Medicine Research Institute, Mona, Kingston 7

Tel: 927-2471; Fax: 927-2984

Email: jennifer.knight-madden@uwimona.edu.jm

Short summary

Children with sickle cell disease (SCD) are at risk of various complications, many of which arise suddenly and call on the problem solving skills of their parents. They are also at risk of neurocognitive delays; these may become evident during the pre-school years. Previous research by this group has demonstrated improved developmental outcomes after parental interventions incorporating play techniques in high risk and healthy pre-school children. The aim of the study is to determine usefulness in children with SCD of similar developmental interventions augmented by lessons in problem solving.

The study is a randomized controlled trial. Parents of all infants with Hb SS or Sβ^0-thalassemia disease identified by newborn screening during the initial year of the study will be invited to participate. They will be randomized to intervention or control. Parental stress, infant's development and hemoglobin levels will be measured at recruitment and again after six months. The intervention will occur during monthly penadur visits and will include supervised play with their children using an inexpensive toy and participation in solving a problem which may arise as they parent their child with SCD. At the end of the study, all control dyads will also be given the toys used in the intervention. All parents will be given the results of their children's developmental assessments and appropriate referrals made if deficits are identified.

The study is minimal risk. Every effort will be made to maintain patient confidentiality. Respect for, and the maximum protection of the best interests of the research subjects will be maintained.

Introduction

The Child Development Research Group (CDRG) of Tropical Medicine Research Institute (TMRI) has pioneered the use of simple, cost effective interventions in the improvement of development in young at risk children. The Sickle Cell Unit (SCU) has provided care for children with sickle cell disease (SCD) for decades, concurrently pursuing many research projects aimed at improving their lives. This project seeks to combine the strengths of these two groups.

SCD was declared by the United Nations and World Health Organization to be a global public health problem (1), with more than 300,000 affected infants born annually (2). SCD is a public health issue in Jamaica where 1:150 babies are born annually with the condition. Neurocognitive deficits have been demonstrated in pre-school children with SCD (3), even in the absence of cerebrovascular accidents (4-7). Educational delays have also been demonstrated in school-aged children (8, 9). Rehabilitative interventions have been attempted, without much success, in those who already have demonstrated deficits. Reduction in educational attainment impacts on vocational and employment outcomes, and the ability of affected persons to become self-sufficient, productive members of society. In most affected persons, SCD precludes strenuous vocations, so the maximizing of academic potential is critical.

The important risk factors for poor early cognitive development are disease severity, as indicated by anaemia, and increased parental stress (5, 10, 11). Parental stress is also higher in parents/caregivers of children with a chronic illness (12), and can worsen both child and adult outcomes (13). Interventions available in developed countries, such as chronic transfusion and hydroxyurea, which can attenuate anaemia, are not sustainable in the developing world. Integration of an intervention into usual care with little additional cost can have significant impact on maximizing the potential of children given their disease severity.

We have demonstrated benefits to child development in at risk groups of an intervention improving parent-child interactions through play, delivered during home visits by health workers, and more recently in a clinic setting. In addition, problem solving therapy (PST), which is based on cognitive behavioural therapy model, has been shown to be effective in persons with depression, and caregivers of children with chronic illness (14-16). In children with SCD, enhanced problem solving skills in the parents have shown an association with improvements in the child’s quality of life(17). Parental education (17), income and family functioning (13) have been shown have correlations with their problem solving skills and stress levels.

We therefore propose to assess the efficacy of these two interventions, delivered during routine monthly clinic visits, in improving parent psychological outcomes and child development. Our hypothesis is that this parenting intervention will improve maternal and child outcomes.

Our specific hypotheses are:

1. Intervention using parent-child interaction through play improve child developmental scores
2. Intervention using parent-child interaction through PST will improve parental outcomes such as stress, coping, and their problem solving skills

Methods

The Study Population

All parents of children aged 6-9 months with severe SCD genotypes (Hb SS disease and Hb Sβ^0 thalassemia) attending the Sickle Cell Unit at Mona Campus, UWI will be informed of the study and given an opportunity to participate. Once the written, informed consent process has been completed, the parent-child dyad will be randomly allocated to intervention or control arms using a random number table. The control dyads will receive usual clinical care.

Procedures

A study coordinator, who is not involved in anyway directly with clinical care of persons with SCD, will approach the parent/caregiver either at the SCU while they are waiting to be seen by their physician/ or via telephone contact. The purpose and the procedures for the study will be thoroughly explained to them and all queries will be addressed. The concept of randomization to intervention or control will be explained by the study coordinator during the informed consent process.
Each dyad will have 8 study visits:

- 1 Entry visit (T0)
- 6 Intervention visits
- 1 Exit visit (T1)

As the two genotypes (SS and Sβ^0 thalassemia) may be indistinguishable clinically as well as haematologically at initial screening of baby at entry to care at SCU, the genotypes are usually confirmed at 9-12 months of age when their HbA2 and HbF levels stabilize and hence assist to confirm the diagnosis. This is done free of cost to the patient. These data will be used to assess anemia in the study. No additional blood tests will be required.

Assessment

Assessments will be done at baseline (T0) and six months later, after completion of the intervention (T1), and will include:

- Assessment of development status of the child,
- Assessment of parent problem solving skills and , stress, and coping.

All assessments will be conducted by trained testers (who are blinded to the randomization arm of the participant) and all questionnaires will be interviewer administered in private surroundings. All questionnaires will be pilot tested prior to commencement of the study with parent/child dyads not included in the study. It is anticipated that the time for completion of assessments and questionnaires will be 60-90 minutes.

Instruments

Social Problem-Solving Inventory–Revised (SPSI-R): This is a 25-item instrument for use in persons over 13 years of age. It provides a total (SPSI-R-Total), as well as the following 5 major scale scores:

- Positive Problem Orientation (PPO)

- Negative Problem Orientation (NPO)

- Rational Problem Solving (RPS)
- Impulsive/Careless Style (ICS)
- Avoidance Style (AS)

It has been used in studies across a variety of clinical conditions including Cancer, Diabetes, caregivers of persons and children with chronic diseases, etc. Email has been sent to distributors of this instrument seeking permission and/or applying for license to use.

Pediatric Inventory for Parents (PIP): Was developed by Streisand in2001 and is one of the commonest used instruments to examine perceived stress in parents who have a child with an illness. It is a 42-item instrument scored on a Likert scale, and has questions regarding how often a particular event has occurred and how difficult that event was for the them. A total score and scores in 4 domains (Communication, Medical care, Emotional Disturbances, and Role Functioning) can be calculated for each area. The scale has been used widely in parents/caregivers of children with cancer, diabetes, and sickle cell disease. Permission for use has been granted by its author (See Appendix 1).

Coping Health Inventory for Parents (CHIP): a 45-item measure developed in 1983 and aims to measures how parent/s or caregiver/s cope while parenting/ managing a child with a chronic illness (See Appendix 2). It is scored on a Likert scale and has 3 dimensions:

1. Maintaining family integration, cooperation, and an optimistic definition of the situation

2. Maintaining social support, self-esteem, and psychological stability

3. Understanding the medical situation through communication with other parents and consultation with medical staff.

Email has been sent to distributors of this instrument seeking permission and applying for license to use.

Griffiths Mental Development Scales: This instrument measures infant's overall development (DQ) and specifically gross and fine motor development, language and cognition. Originally developed by Ruth Griffith in 1954, the revised version published by Hogrefe (1996) will be used. After adapting this test for children in Jamaica we have used it in numerous studies. The instrument has been found to be sensitive in showing differences between nutritionally deprived children and adequately nourished children as well as showing effects of interventions (Grantham-McGregor SM, Powell CA, Walker SP, Himes JH. Nutritional supplementation, psychosocial stimulation, and mental development of stunted children: the Jamaican Study. *Lancet* 1991; **338:** 1–5.). In Jamaica, the Griffiths correlated well with later tests of intelligence and academic achievement. (Grantham-McGregor S, Powell C, Walker S, Chang S, Fletcher P. The long-term follow-up of severely malnourished children who participated in an intervention program. Child Dev. 1994 Apr;65:428-39.)

Intervention

<u>Interventions will be undertaken in a private room, away from the general clinic. This will avoid leakage of the intervention to the control group.</u> Both interventions, i.e. Problem-Solving

Therapy (PST) and Play Therapy, will be conducted by clinic nurses who will undergo an initial period of training in each intervention with the study investigators. These interventions will be done during monthly clinic visits when the babies must attend for receiving penadur injections for infection prophylaxis. The interventions will be done with small groups of parents and will take no longer than an hour.

Play Therapy

Short films that have been developed to deliver a series of child development messages will be shown at the monthly visits. Nine modules of approximate length 3 minutes each have been developed and cover the topics: Love, Comforting baby, Talking to babies and children, Praise, Using bath time to play and learn, Looking at books, Simple toys parents can make, Drawing and games, and Puzzles. Three topics will be shown together at each visit, allowing each topic to be shown on more than one occasion. Nurses at the SCU will be trained to discuss the video messages with the parents/caregivers, demonstrate activities they can do with their children and how to make simple toys from household materials. Opportunities will be given for parents/caregivers to try some of the activities and they will be encouraged to make them part of their daily routine.

PST

Problem solving therapy aims to empower patients or caregivers in attending to daily social and other challenges that might arise especially with the presence of a chronic illness. The approach is based on cognitive behavioural therapy and has been shown to be of use in primary care settings (15, 16, 18, 19). It encourages persons to use existing resources and skills to function better and find solutions to problems (20). It will be delivered in 6 sessions over the 6-week period and will last for about 30 minutes per session. The stages of PST are: identification of the problems; generating possible solutions; evaluating and implementing preferred solution; and evaluating to see if the solutions were successful (14, 18). PST has been used effectively in various stress related conditions, anxiety, and stress.

The parent/caregiver will be taught a process of problem solving with reference to general everyday problems as well as specific problems which may arise while parenting a child with SCD.

Sample size and statistical considerations

A Jamaican trial of home visiting parent intervention integrated into primary care demonstrated an effect size of 0.8 SD (21). Due to the smaller number of contacts with parents in the new intervention, we hypothesized an effect size of 0.67 SD. A sample comprising 35 children per group achieves 80% power to detect the hypothesized effect.

All analyses will be performed using the statistical software STATA 12.0, based on intention-to-treat principles. Descriptive statistics (means ± SD, or median and interquartile ranges, as appropriate) will be used to describe the study sample with regards to baseline characteristics. Comparisons of primary and secondary outcomes at both T0 and T1 between the intervention

and control groups will be analyzed. When relevant the change in outcomes from baseline to follow-up will be assessed. Statistical significance is being set at $P < 0.05$.

Confidentiality Procedures

Confidentiality of all participants will be protected within legal limits. Each parent-child dyad will be assigned a study number and all relevant clinical and experimental data will be stored in notebooks and computers at the SCU under the assigned number. Access to data will be limited to those personnel directly involved in the study.

Benefits &Risks to the participants

There is no payment for participation. However, **we will transport the dyads to clinic for the initial and final assessments. This is necessary because the Child Development Research Groups has found that children who travel on Jamaican public transportation do not perform optimally in developmental tests. We also will pay bus fare, a maximum of J$2,500 but based on where the dyad lives, for the six visits during the intervention.**

If the intervention is useful, parents may benefit from improved mental health and infants improve in development. Furthermore, these parents will be able to apply the skills they have learned to their relationships with other children. After the second assessment, all parents will be given the results of their children's developmental assessments and appropriate referrals made if deficits are identified. At the end of the study, all control dyads will also be given the toys used in the intervention. If the intervention demonstrates benefit, the control dyads will have it delivered as a group.
This is a proof of concept/pilot study. Were it to show benefit, the intervention could be developed further with the SCD Working Group lead by the Ministry of Health to determine whether it is feasible to scale up in the future.

The study will present minimal risks to the participants beyond those associated with blood sampling, which will be conducted by trained medical technologists. If any of the questions cause any emotional discomfort to the participants, they will be referred for appropriate counselling by the social worker at SCU.

References

1. World Health Organization. Recognition of sickle cell anaemia as a public health problem. Geneva: Sixty-Third Session. A/RES/63/237; 2009. p. 1-2.

2. Weatherall DJ. The inherited diseases of hemoglobin are an emerging global health burden. Blood. 2010;115(22):4331-6. Epub 2010/03/18.

3. Schatz J, McClellan CB, Puffer ES, Johnson K, Roberts CW. Neurodevelopmental screening in toddlers and early preschoolers with sickle cell disease. Journal of child neurology. 2008;23(1):44-50. Epub 2007/12/28.

4. Brown RT, Armstrong FD, Eckman JR. Neurocognitive aspects of pediatric sickle cell disease. Journal of learning disabilities. 1993;26(1):33-45. Epub 1993/01/01.

5. Thompson RJ, Jr., Gustafson KE, Bonner MJ, Ware RE. Neurocognitive development of young children with sickle cell disease through three years of age. Journal of pediatric psychology. 2002;27(3):235-44. Epub 2002/03/23.

6. Wasserman AL, Wilimas JA, Fairclough DL, Mulhern RK, Wang W. Subtle neuropsychological deficits in children with sickle cell disease. The American journal of pediatric hematology/oncology. 1991;13(1):14-20. Epub 1991/01/01.

7. Steen RG, Fineberg-Buchner C, Hankins G, Weiss L, Prifitera A, Mulhern RK. Cognitive deficits in children with sickle cell disease. Journal of child neurology. 2005;20(2):102-7. Epub 2005/03/30.

8. Epping AS, Myrvik MP, Newby RF, Panepinto JA, Brandow AM, Scott JP. Academic attainment findings in children with sickle cell disease. The Journal of school health. 2013;83(8):548-53. Epub 2013/07/10.

9. Schatz J. Brief report: Academic attainment in children with sickle cell disease. Journal of pediatric psychology. 2004;29(8):627-33. Epub 2004/10/20.

10. Quinn CT, Dowling MM. Cerebral tissue hemoglobin saturation in children with sickle cell disease. Pediatric blood & cancer. 2012;59(5):881-7. Epub 2012/06/09.

11. Tarazi RA, Grant ML, Ely E, Barakat LP. Neuropsychological functioning in preschool-age children with sickle cell disease: the role of illness-related and psychosocial factors. Child

neuropsychology : a journal on normal and abnormal development in childhood and adolescence. 2007;13(2):155-72. Epub 2007/03/17.

12. Cousino MK, Hazen RA. Parenting stress among caregivers of children with chronic illness: a systematic review. Journal of pediatric psychology. 2013;38(8):809-28. Epub 2013/07/12.

13. Barakat LP, Patterson CA, Weinberger BS, Simon K, Gonzalez ER, Dampier C. A prospective study of the role of coping and family functioning in health outcomes for adolescents with sickle cell disease. Journal of pediatric hematology/oncology. 2007;29(11):752-60. Epub 2007/11/07.

14. Eccleston C, Palermo TM, Fisher E, Law E. Psychological interventions for parents of children and adolescents with chronic illness. The Cochrane database of systematic reviews. 2012;8:CD009660. Epub 2012/08/17.

15. Hassink-Franke LJ, van Weel-Baumgarten EM, Wierda E, Engelen MW, Beek MM, Bor HH, et al. Effectiveness of problem-solving treatment by general practice registrars for patients with emotional symptoms. Journal of primary health care. 2011;3(3):181-9. Epub 2011/09/06.

16. Law EF, Fisher E, Fales J, Noel M, Eccleston C. Systematic Review and Meta-Analysis: Parent and Family-Based Interventions for Children and Adolescents With Chronic Medical Conditions. Journal of pediatric psychology. 2014. Epub 2014/06/02.

17. Barakat LP, Daniel LC, Smith K, Renee Robinson M, Patterson CA. Parental problem-solving abilities and the association of sickle cell disease complications with health-related quality of life for school-age children. Journal of clinical psychology in medical settings. 2014;21(1):56-65. Epub 2013/11/14.

18. Franke LJ, van Weel-Baumgarten EM, Lucassen PL, Beek MM, Mynors-Wallis L, van Weel C. Feasibility of training in problem-solving treatment for general practice registrars. The European journal of general practice. 2007;13(4):243-5. Epub 2008/03/08.

19. Huibers MJ, Beurskens AJ, Bleijenberg G, van Schayck CP. The effectiveness of psychosocial interventions delivered by general practitioners. The Cochrane database of systematic reviews. 2003(2):CD003494. Epub 2003/06/14.

20. Mynors-Wallis LM, Gath DH, Day A, Baker F. Randomised controlled trial of problem solving treatment, antidepressant medication, and combined treatment for major depression in primary care. Bmj. 2000;320(7226):26-30. Epub 2000/01/05.

21. **Powell C, Baker-Henningham H, Walker S, Gernay J, Grantham-McGregor S. Feasibility of integrating early stimulation into primary care for undernourished Jamaican children: cluster randomised controlled trial. BMJ. 2004 Jul 10;329:89.**

Consent Form

TITLE OF PROTOCOL:

Integrating a parenting intervention with routine care to decrease parental stress and improve early developmental outcomes in children with sickle cell disease

PURPOSE OF THE STUDY

Children with sickle cell disease (SCD) are at risk of various complications, many of which arise suddenly and which may require the parent to make quick and correct decisions. They are also at risk of delays in their development. There are many techniques that have helped other children's development and parents' coping skills. We wish to see if some of these methods will assist both the child with SCD and their families.

This study is being undertaken to determine how having a baby with SCD might affect you the parent emotionally, how well you are coping with and managing any challenges. We also wish to understand the baby's development and see whether some new activities at the clinic might help parents levels of stress and coping capabilities as well as the baby' development .

PROCEDURES

You are being asked to participate in this study because you have just had a new baby who has been diagnosed with SCD. If you decide to participate, you will be asked to complete a questionnaire, which asks various questions about how you are feeling emotionally, how well you are coping, and how well you are able to face challenges. We will also be doing a test, using toys, on your baby to see how s/he is developing. The questionnaire and testing will take about 60-90 minutes of your time for completion. Even though your I.D. number will be seen on the questionnaire, your baby's or your identity will never be disclosed and strict confidence will be maintained.

Some of the parents who take part in the study will be asked to join us for some new sessions once per month for the next six months during the regular monthly visits to the clinic for the baby's penadur injections. Other parents will come to the clinic for the regular monthly visits as usual without these new sessions. The group in which you will be in is determined randomly and at the end of the interview period. If you are in the group with sessions, we will have discussions and demonstrations of ways to help young babies develop well. It will include showing you short videos on ways to interact with your child. We will also discuss ways to tackle any problems or challenges you might encounter and your own experiences. Each session is expected last about 1 hour.

After the 6 months you will be asked to attend the clinic to complete the same questionnaire again and the baby's development will also be reassessed.

After the second assessment, all parents will be given the results of their children's developmental assessments and appropriate referrals made if deficits are identified. If you are in the group who did not have sessions, you will be given the toys used in the intervention at the end of the study. Also, if the intervention demonstrates benefit, you will have the opportunity to attend group sessions where we will show the videos and discuss problem solving.

The results of the blood test which are routinely done by the SCU at no cost to you to confirm the type of sickle cell disease your child has will be used by us to check whether anemia affects development. This is done when your baby is 9-12 months of age.

BENEFITS AND RISKS

There will be no financial benefits to you if you agree to participate in this study. We **will transport you and your infant to clinic for the initial and final assessments. This is necessary so that your infant will not be tired when doing the developmental tests. We also will reimburse bus fare, based on where you live, for the six visits during the intervention.** The results of this study will assist in determining if there are benefit of these techniques and whether they should be offered to other children and families. There are no physicals risks to you as a result of this study. However if any of the questions cause any emotional discomfort, you can be referred for appropriate counselling by the social worker at SCU at no cost to you.

ALTERNATIVES

You do not have to participate in this study if you do not want to do so. Also, you can decide at any time during the study that you wish to withdraw. If you do not participate, or withdraw during the study, there will be no change in your present or future care at the Sickle Cell Unit or the University Hospital of the West Indies.

If there are any questions, the study staff will try their best to answer them to your satisfaction. If you have any questions regarding the research project, you may contact any of the Principal Investigators: Dr. Jennifer Knight-Madden or Dr. Susan Chang-Lopez at 927-2471 or email them at Jennifer.knightmadden@uwimona.edu.jm or susan.changlopez@uwimona.edu.jm. For independent advice on your rights as a research participant you may contact Prof. Horace Fletcher, Dean, Faculty of Medical Sciences, UWI, Mona, and Kgn.7 at Tel: (876) 927-1297 or email him at medsci@uwimona.edu.jm.

STATEMENT OF DECLARATION

I have read the information provided above and/or it has been read and explained to me. All my questions have been answered to my satisfaction. My signature below indicates that I give my consent to participate in the study. In so doing, I understand that I can withdraw my consent at any time as I so desire. I also understand that any questions that I may have in the future will be answered willingly and promptly.

Name of Respondent: ______________________

Signature of Respondent: _________________

Date: _______________________________

Name of Interviewer: ______________________

Signature of Interviewer: _________________

Date: _________________________

Signature of Independent Witness: _______________________

Integrating a parenting intervention with routine care to improve parental psychosocial functioning and early developmental outcomes in children with sickle cell disease

This study seeks to determine how parents of a new baby with sickle cell disease cope, how confident they feel in facing challenges of parenting and their emotional feelings; as well as the baby's development. The study will further determine if certain methods can be employed during routine visits to the sickle cell clinic that could improve both the child's development as well as parent's stress and coping capacities.

Please circle the correct response or fill in the blanks where required.

Questionnaire #: _______

Baby's Information

Name of Baby: ________________________________

Baby's SCU ID Number: _________

Genotype of baby: SS / SB0Thal

Height (cm):_________ Weight(Kg):_____________

Sex: Male / Female

D.O.B.: ___/___/_______ Age: __________

dd mm yy

Parent/caregiver Information

Parent/Caregiver name: _______________________

Sex: Male / Female

D.O.B.: ___/___/_______ Age: __________

dd mm yy

Address :

Community: _______________ _________

Parish: ______________________ Town or 'Rural': ______________

A. Socio-Demographics

1. What is the highest level of schooling that you have completed?

   a. No schooling
   b. Primary (Grades 1-6)

c. Secondary (Grades 7-11)
d. Post-Secondary (Grades 12-13)
e. Technical/Vocational
f. University

2. What is your main occupation?

____

3. What is your employment status?

a. Full time *(30 or more hours/week)*
b. Part time *(29 or fewer hours/week)*
c. Unemployed and looking
d. Unemployed and not looking
e. Student

4. What is your current marital status?

a. Never married
b. Married
c. Separated/Divorced
d. Common-Law
e. Visiting relationship

5. Who lives at home with you? *(Tick all that apply)*

Persons who live with respondent:	Yes [1]	No [0]	Number(s)
a.) Spouse/Consort			
b.) Children			
c.) Parent			
d.) Father			
e.) Guardian			
f.) Grandparents/ Great grandparents			
g.) Aunt(s)/Uncle(s)			
h.) Brother(s)			
i.) Sister(s)			
k.) Other Relative(s)			

Persons who live with respondent:	Yes [1]	No [0]	Number(s)
l.) Other non-relative(s)			
Total no. of persons *(Exclude respondent)*			

6. How many rooms do you have inside your house? (Count Bedrooms, bathrooms, kitchen, living room, dining room, study room separately)

_________ Rooms

7. How many of the following possessions do you have in your house?

________ Items

1. Sewing machine
2. Gas stove/ Electric stove
3. Refrigerator or freezer
4. Microwave oven
5. Air conditioner
6. Fan
7. Telephone
8. Radio/cassette player
9. Stereo equipment/component set
10. Compact Disk/CD player
11. Video cassette recorder/VCR/DVD
12. Washing machine
13. TV set
14. Cable TV
15. Satellite dish/DSS
16. Bicycle
17. Motorbike
18. Car, other vehicle
19. Computer/Printer/Fax, etc.
20. Internet access

8. What type of toilet facilities do you have? *(Tick all relevant options)*

a. None
b. Pit Latrine
c. Flush toilet

9. What is your main source of water for drinking? *(Tick all relevant options)*

a. River/Spring
b. Tank/Drum
c. Standpipe
d. Pipe inside house
e. Bottled water

10. By what means have you been able to access and use the internet?*(Tick all relevant options)*

a. On computer at home

b. On a smart phone

c. At school

d. At public library

e. At community centre

f. No access to internet

g. Other (explain) ____

Other Questionnaires

B. PIP

C. CHIP

D. SPSI-R

Appendix 1

Dear Colleague,

Thank you for your interest in the Pediatric Inventory for Parents. Included in this e-mail are the measure and scoring instructions. I grant you permission to use the measure in your work. Please keep me informed of any results as your work progresses, and feel free to contact me with any further questions.

In addition to the measure you will also find scoring instructions attached. Further, attached are references from investigations that have included the PIP, following the initial article from 2001*.

Best wishes on your research,

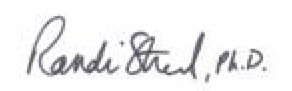

Randi Streisand, PhD, CDE
Diabetes Team Director of Psychology Research and Service
Children’s National Medical Center
Associate Professor of Psychiatry and Pediatrics,
The George Washington University
(202) 884-2730
rstreis@cnmc.org

PEDIATRIC INVENTORY FOR PARENTS

Below is a list of difficult events which parents of children who have (or have had) a serious illness sometimes face. Please read each event carefully, and circle HOW OFTEN the event has occurred for you <u>in the past 7 days</u>, using the 5 point scale below. Afterwards, please rate how DIFFICULT it was/or generally is for you, also using the 5 point scale. <u>Please complete both columns for each item.</u>

EVENT	HOW OFTEN?<br>1=Never,<br>2=Rarely,<br>3=Sometimes,<br>4=Often,<br>5=Very often	HOW DIFFICULT?<br>1=Not at all,<br>2=A little,<br>3=Somewhat,<br>4=Very much,<br>5=Extremely
1. Difficulty sleeping	1 2 3 4 5	1 2 3 4 5
2. Arguing with family member(s)	1 2 3 4 5	1 2 3 4 5
3. Bringing my child to the clinic or hospital	1 2 3 4 5	1 2 3 4 5
4. Learning upsetting news	1 2 3 4 5	1 2 3 4 5
5. Being unable to go to work/job	1 2 3 4 5	1 2 3 4 5
6. Seeing my child's mood change quickly	1 2 3 4 5	1 2 3 4 5
7. Speaking with doctor	1 2 3 4 5	1 2 3 4 5
8. Watching my child have trouble eating	1 2 3 4 5	1 2 3 4 5
9. Waiting for my child's test results	1 2 3 4 5	1 2 3 4 5
10. Having money/financial troubles	1 2 3 4 5	1 2 3 4 5
11. Trying not to think about my family's difficulties	1 2 3 4 5	1 2 3 4 5
12. Feeling confused about medical information	1 2 3 4 5	1 2 3 4 5
13. Being with my child during medical procedures	1 2 3 4 5	1 2 3 4 5
14. Knowing my child is hurting or in pain	1 2 3 4 5	1 2 3 4 5
15. Trying to attend to the needs of other family members	1 2 3 4 5	1 2 3 4 5
16. Seeing my child sad or scared	1 2 3 4 5	1 2 3 4 5
17. Talking with the nurse	1 2 3 4 5	1 2 3 4 5
18. Making decisions about medical care or medicines	1 2 3 4 5	1 2 3 4 5
19. Thinking about my child being isolated from others	1 2 3 4 5	1 2 3 4 5
20. Being far away from family and/or friends	1 2 3 4 5	1 2 3 4 5
21. Feeling numb inside	1 2 3 4 5	1 2 3 4 5

EVENT	HOW OFTEN? 1=Never, 2=Rarely, 3=Sometimes, 4=Often, 5=Very often	HOW DIFFICULT? 1=Not at all, 2=A little, 3=Somewhat, 4=Very much, 5=Extremely
22. Disagreeing with a member of the health care team..................	1 2 3 4 5	1 2 3 4 5
23. Helping my child with his/her hygiene needs............................	1 2 3 4 5	1 2 3 4 5
24. Worrying about the long term impact of the illness	1 2 3 4 5	1 2 3 4 5
25. Having little time to take care of my own needs	1 2 3 4 5	1 2 3 4 5
26. Feeling helpless over my child's condition	1 2 3 4 5	1 2 3 4 5
27. Feeling misunderstood by family/friends as to the severity of my child's illness	1 2 3 4 5	1 2 3 4 5
28. Handling changes in my child's daily medical routines............	1 2 3 4 5	1 2 3 4 5
29. Feeling uncertain about the future	1 2 3 4 5	1 2 3 4 5
30. Being in the hospital over weekends/holidays...........................	1 2 3 4 5	1 2 3 4 5
31. Thinking about other children who have been seriously ill.......	1 2 3 4 5	1 2 3 4 5
32. Speaking with my child about his/her illness	1 2 3 4 5	1 2 3 4 5
33. Helping my child with medical procedures (e.g. giving shots, swallowing medicine, changing dressing)..................................	1 2 3 4 5	1 2 3 4 5
34. Having my heart beat fast, sweating, or feeling tingly..............	1 2 3 4 5	1 2 3 4 5
35. Feeling uncertain about disciplining my child...........................	1 2 3 4 5	1 2 3 4 5
36. Feeling scared that my child could get very sick or die.............	1 2 3 4 5	1 2 3 4 5
37. Speaking with family members about my child's illness	1 2 3 4 5	1 2 3 4 5
38. Watching my child during medical visits/procedures................	1 2 3 4 5	1 2 3 4 5
39. Missing important events in the lives of other family members	1 2 3 4 5	1 2 3 4 5
40. Worrying about how friends and relatives interact with my child	1 2 3 4 5	1 2 3 4 5
41. Noticing a change in my relationship with my partner..............	1 2 3 4 5	1 2 3 4 5
42. Spending a great deal of time in unfamiliar settings	1 2 3 4 5	1 2 3 4 5

Appendix 2

Coping Health Inventory for Parents (CHIP)

Instructions to be given by interviewer

I will be reading out a list of possible coping behaviours.

For each coping behaviour you used, please record how helpful it was. The options are:

3 Extremely helpful

2 Moderately helpful

1 Minimally helpful

0 Not helpful

For each coping behaviour (CB) you did not use please record your reason . The options are :

A. "Chose not to use it"

B. "Not possible."

Scale items:

1. Believing that my child(ren) will get better.
2. Investing myself in my children.
3. Doing things with my children.
4. Believing that things will always work out.
5. Telling myself that I have many things I should be thankful for.
6. Building a closer relationship with my spouse.
7. Talking over personal feelings and concerns with spouse.
8. Doing things with family relatives.
9. Believing in God.
10. Taking good care of all the medical equipment at home.

11. Believing that my child is getting the best medical care possible.
12. Trying to maintain family stability.
13. Doing things together as a family (involving all members of the family).
14. Trusting my spouse (or former spouse) to help support me and my child(ren).
15. Showing that I am strong.
16. Getting other members of the family to help with chores and tasks at home.
17. Having my child with the medical condition seen at the clinic/hospital on a regular basis.
18. Believing that the medical centre/hospital has my family's best interest in mind.
19. Encouraging child(ren) with medical condition to be more independent.
20. Involvement in social activities (parties, etc.) with friends.
21. Being able to get away from the home care tasks and responsibilities for some relief.
22. Getting away by myself.
23. Eating.
24. Sleeping.
25. Allowing myself to get angry.
26. Purchasing gifts for myself and/or other family members.
27. Concentrating on hobbies (art, music, jogging, etc.).
28. Working, outside employment.
29. Becoming more self-reliant and independent.
30. Keeping myself in shape and well-groomed.
31. Talking to someone (not professional counsellor/doctor) about how I feel.
32. Engaging in relationships and friendships which help me to feel important and appreciated.
33. Entertaining friends in our home.
34. Investing time and energy in my job.
35. Going out with my spouse on a regular basis.
36. Building close relationships with people.
37. Developing myself as a person.
38. Talking with other parents in the same type of situation and learning about their experiences.
39. Talking with the medical staff (nurses, social worker, etc.) when we visit the medical center.

40. Reading about how other persons in my situation handle things.

41. Reading more about the medical problem which concerns me.

42. Explaining our family situation to friends and neighbours so they will understand.

43. Being sure prescribed medical treatments for child(ren) are carried out at home on a daily basis.

44. Talking with other individuals/parents in my same situation.

45. Talking with the doctor about my concerns about my child(ren) with the medical condition.

USE THE FOLLOWING RESPONSE CATEGORIES FOR SCALES ABOVE.
